# Supplementary material for: Asymmetric Synthesis of Quaternary Hydantoins via a Palladium-Catalyzed Aza-Heck Cyclization
Source: J Am Chem Soc. 2025 Nov 14;147(49):44692–8. doi: 10.1021/jacs.5c16022 (PMC12703750; doi:10.1021/jacs.5c16022)
Supplement: Supplementary file 2 [file ja5c16022_si_002.zip › All NMR FID Files/S5/S5_ProtonNMR/1/pdata/1/email_EM06-129-1-F21-63-1H_1_1.pdf]

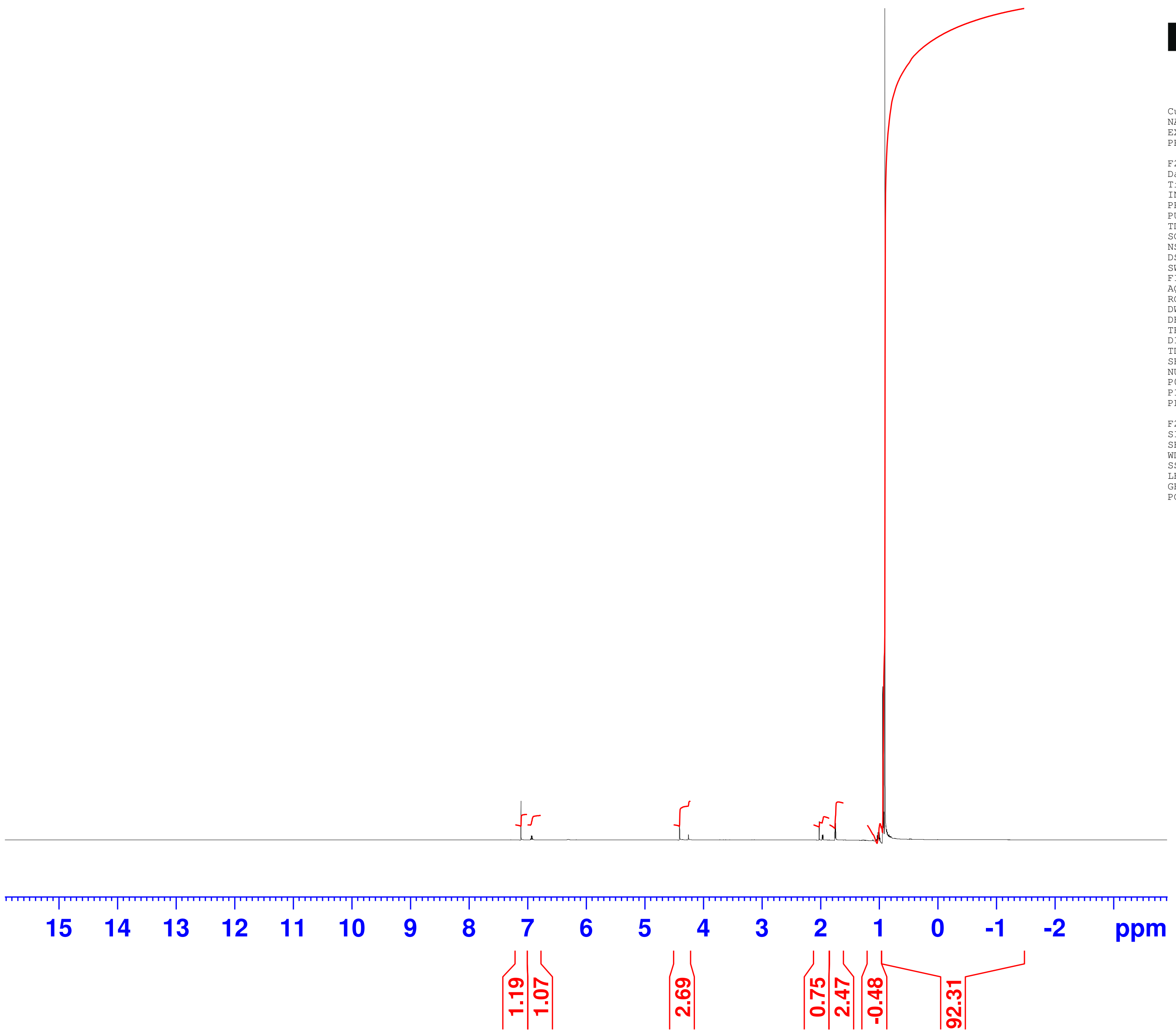

BRUKER

Current Data Parameters
NAME EM06-129-1-F21-63-1H
EXPNO 1
PROCNO 1

F2 - Acquisition Parameters
Date_ 20250311
Time 20.45 h
INSTRUM Avance Neo 600 at University of Delaware
PROBHD Z185170_0001 (
PULPROG zg30
TD 65536
SOLVENT CDCl3
NS 16
DS 2
SWH 11904.762 Hz
FIDRES 0.363304 Hz
AQ 2.7525120 sec
RG 18
DW 42.000 usec
DE 14.37 usec
TE 298.0 K
D1 1.00000000 sec
TD0 1
SFO1 600.1337058 MHz
NUC1 1H
P0 2.69 usec
P1 8.07 usec
PLW1 6.90000010 W

F2 - Processing parameters
SI 65536
SF 600.1301041 MHz
WDW EM
SSB 0
LB 0.30 Hz
GB 0
PC 1.00
